# Correction: A two-lane mechanism for selective biological ammonium transport

**DOI:** 10.7554/eLife.77377

**Published:** 2022-01-27

**Authors:** Gordon Williamson, Giulia Tamburrino, Adriana Bizior, Mélanie Boeckstaens, Gaëtan Dias Mirandela, Marcus G Bage, Andrei Pisliakov, Callum M Ives, Eilidh Terras, Paul A Hoskisson, Anna-Maria Marini, Ulrich Zachariae, Arnaud Javelle

**Keywords:** *E. coli*

Williamson G, Tamburrino G, Bizior A, Boeckstaens M, Mirandela GD, Bage MG, Pisliakov A, Ives CM, Terras E, Hoskisson PA, Marini AM, Zachariae U, Javelle A. 2020. A two-lane mechanism for selective biological ammonium transport. *eLife* **10**:e57183. doi: 10.7554/eLife.57183

Published 14 July 2020

In Figure 4 panel B, we inadvertently used the same image to represent the lack of yeast growth for both D160A and D160E variants of AmtB. This has been corrected and D160E now has the appropriate image. As both the original and corrected panel show the same result, the text and figure legend remain unchanged.

The article has been corrected accordingly.

