# Supplementary material for: A two-lane mechanism for selective biological ammonium transport
Source: eLife. 2020 Jul 14;9:e57183. doi: 10.7554/eLife.57183 (PMC7447429; doi:10.7554/eLife.57183)
Supplement: Supplementary file 1. [file elife-57183-supp1.docx]

**A two-lane mechanism for selective biological ammonium transport**

Gordon Williamson, Giulia Tamburrino, Adriana Bizior, Mélanie Boeckstaens_,_ Gaëtan Dias Mirandela, Marcus Bage, Andrei Pisliakov, Callum M. Ives, Eilidh Terras, Paul A. Hoskisson, Anna Maria Marini, Ulrich Zachariae and Arnaud Javelle

## **Supplementary tables**

## **Supplementary Table 1**: Solid-Supported Membrane Electrophysiology Solutions*

| **Substrate** | **Activating** | **Non-Activating** |
| --- | --- | --- |
| NH_4_^+^ | 100 mM KPho | 100 mM KPho |
|  | 100 mM KCl | 300 mM KCl |
|  | 200 mM NH_4_Cl |  |
| H^+^ | 100 mM KPho | 100 mM KPho |
|  | 300 mM KCl | 300 mM KCl |
|  | pH5 | pH8 |
| K^+^ | 100 mM NaPho | 100 mM NaPho |
|  | 100 mM NaCl | 300 mM NaCl |
|  | 200 mM KCl |  |

*All solutions adjusted to pH 5, 7 or 8 as required. KPho: potassium phosphate buffer, NaPho: sodium phosphate buffer. For the D_2_O experiments, all the solutions were prepared using D_2_O instead of water.
